# Protocol to measure centromeric array size changes using droplet digital PCR-based quantification of higher-order repeats

**DOI:** 10.1016/j.xpro.2024.103218

**Published:** 2024-07-27

**Authors:** Soyeon Showman, Paul B. Talbert, Yiling Xu, Steven Henikoff

**Affiliations:** 1 Basic Sciences Division, Fred Hutchinson Cancer Center, Seattle, WA 98109, USA; 2 Molecular and Cellular Biology Graduate Program, University of Washington, Seattle, WA 98195, USA; 3 Howard Hughes Medical Institute, Chevy Chase, MD 20815, USA

**Keywords:** Genomics, Molecular Biology, Evolutionary biology

## Abstract

Centromere length changes occurring during somatic cell divisions can be estimated by quantifying the copy numbers (CNs) of higher-order repeats (HORs), which are nested repeats of monomers that comprise centromeric arrays. Here, we present a protocol for single-cell isolation for clonal evolution followed by droplet digital PCR-based quantification. The assay measures HOR CNs across subclones to determine the frequency and degree of changes in HOR CNs. This protocol tests the underlying molecular mechanisms responsible for rapid centromere sequence evolution.

For complete details on the use and execution of this protocol, please refer to Showman et al.^1^

## Highlights

• Instructions for isolating single cells for clonal evolution
• Procedures for the quantification and preparation of ddPCR samples
• Steps for ddPCR technique to measure centromeric array size changes

## Abstract (editor highlights)

Publisher’s note: Undertaking any experimental protocol requires adherence to local institutional guidelines for laboratory safety and ethics.

## Graphical abstract

**Figure:**
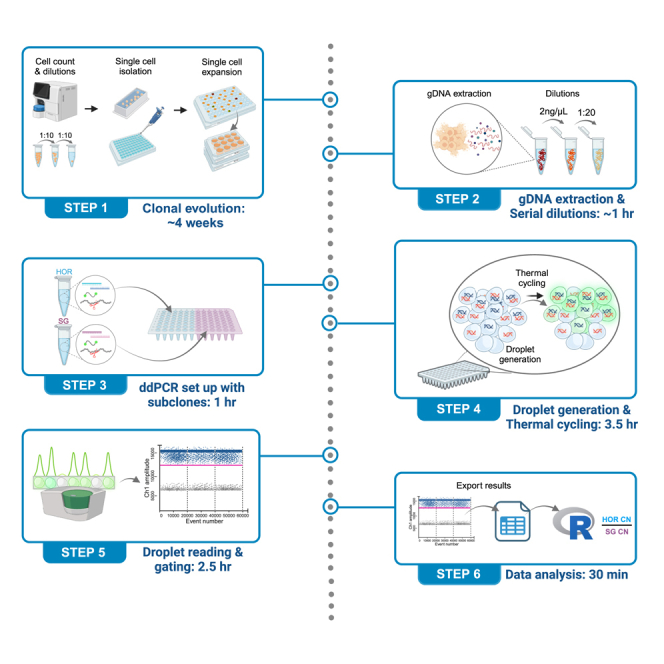

## Before you begin

This protocol aims to quantify CNs of HORs in α-satellite arrays that can change during somatic cell divisions. The protocol below describes the specific steps for measuring D11Z1 CN in human U2OS cells, but we have also applied it to different HORs and other cell lines including human K562 cells with modification during monoclonal cell isolation. This protocol takes 10–12 h of hands-on time spread across 8 weeks to allow for clonal evolution. We recommend using cells that have undergone few sub-cultures to ensure consistent cell growth throughout clonal evolution. This protocol can be used to quantify other repetitive elements, yet optimizations including primer efficiency, genomic DNA (gDNA) input, dilution factor, and annealing temperature are likely needed. We used the human CHM13 cell line for optimization and as a positive control,^2^^,^^3^ yet this can be customized for the tandem repetitive array of interest. This protocol does not provide primer design steps because each repetitive array needs extensive troubleshooting to determine whether the designed primers efficiently target all repeats. All primer sequences that are compatible with this protocol are from de Lima et al., (2021) and are listed in Table 1.^4^

**Table 1:** Target primer sequences and restriction enzymes

Primer sequence	Target (HOR or single gene)	Restriction enzyme
D6Z1 F: 5′ – GCGTTGAACTCACCGTCTT – 3′	HOR (Chromosome 6)	Alu I
D6Z1 R: 5′ – TCCAAAGAATGCCTCCAAGG – 3′	HOR (Chromosome 6)	Alu I
TBP1 F: 5′ – GATATGAGACTGTGGGTAAGT – 3′	SG (Chromosome 6)	HaeIII
TBP1 R: 5′ – GATCCTTTGAACACCCTAATG – 3′	SG (Chromosome 6)	HaeIII
D11Z1 F: 5′ – CTTCCTTCGAAACGGGTATATCT – 3′	HOR (Chromosome 11)	Alu I
D11Z1 R: 5′ – GCTCCATCAGCAGGATTGT – 3′	HOR (Chromosome 11)	Alu I
C11orf16 F: 5′ – TCCCTGACCATCTGGAAGAA – 3′	SG (Chromosome 11)	Alu I
C11orf16 R: 5′ – TGATTGGCCCTAGCAGAGA – 3′	SG (Chromosome 11)	Alu I
D18Z1 F: 5′ – TGGGAAACGGGATTGTCTTC – 3′	HOR (Chromosome 18)	Alu I
D18Z1 R: 5′ – CTGCTCTACCAAAGGGAATGT – 3′	HOR (Chromosome 18)	Alu I
MRO F: 5′ – TAGTAGGTAACACCGAGTGC – 3′	SG (Chromosome 18)	Alu I
MRO R: 5′ – TCAGGGTTGTCGCAAGTA – 3′	SG (Chromosome 18)	Alu I
DXZ1 F: 5′ – TGATAGCGCAGCTTTGACAC – 3′	HOR (Chromosome X)	HaeIII
DXZ1 R: 5′ – TTCCAACACAGTCCTCCA – 3′	HOR (Chromosome X)	HaeIII
HPRT1 F: 5′ – AAGGTGCTGGTCTCCTTTAC – 3′	SG (Chromosome X)	Alu I
HPRT1 R: 5′ – GCACCAATGATTCTCTCCCT – 3′	SG (Chromosome X)	Alu I

### Monoclonal cell line preparation

**Timing: 4 weeks**

1. Culture population of U2OS cells and collect conditioned media.
  a. Thaw a new early passage stock of frozen U2OS cells and passage the cells at least two times.
  b. Seed 1.5 × 10^6^ U2OS cells in a T-75 flask with 15 mL of media.
  c. Incubate the cells at 37°C for 2–3 days until 80% confluence.
  d. Remove old (conditioned) media from the flask and place in a 50 mL conical tube.
  e. Wash cells in the flask once with 1X PBS.
  f. Trypsinize cells in 3 mL of 0.25% trypsin-EDTA and incubate at 37°C for 5 min.
  g. Add 7 mL of media and break up all clumps of cells into individual cells by pipetting up and down.

***Optional:*** Although conditioned media may be used, avoid using it from cells that are overly confluent due to the potential presence of excess cellular waste.

2. Isolate single cells from the population of U2OS cells by limiting dilution.^5^^,^^6^
  a. Measure the cell number of the homogenized cells with a Vi-cell analyzer.
  b. Add 10^6^ cells/mL in a 1.5 mL microcentrifuge tube (refer to Preparation of HOR CN control).
  c. Dilute the cells to 10^5^ cells/mL in a 1.5 mL microcentrifuge tube containing media and homogenize by pipetting.
  d. Measure the cell number of the 10^5^ cells/mL stock.
  e. Dilute the 10^5^ cells/mL stock solution in 1:10 (10,000 cells/mL) and 1:100 (1,000 cell/mL) using 1.5 mL microcentrifuge tubes containing media.
  f. Filter the conditioned media through a 0.22 μm PES filter.
  g. Seed 50 single cells in a 96-well plate by adding 50 μL of the 1:100 diluted cell solution in 10.5 mL of conditioned media and mix thoroughly by pipetting.
  h. Move the cell-containing media to a reservoir and dispense 100 μL of cell solution into each well of a 96-well plate except for one well on the right bottom corner of the plate (H12). This well will be used to focus the microscope while searching for single cells.
  i. Add 100 μL of the 1:10 cell solution in the bottom right corner well.
  j. Keep the plate undisturbed in an incubator for 7 days.

**CRITICAL:** It is essential to homogenize the cell solution during dilution in order to yield a high number of single cells with minimal doublets.

***Optional:*** A hemocytometer or other cell counter may be used instead of a Vi-cell analyzer.

3. Scan the 96-well plate to find monoclonal cells on the 7^th^ day post isolation.
  a. Focus the microscope using the cells in the bottom right corner well (H12).
  b. Scan the entire plate and mark the wells containing single colonies.
  c. Keep monitoring the marked wells every day at roughly the same time and discard any well containing more than a single colony or that are observed to grow at a different rate than the majority.

**CRITICAL:** Having two cells in a well reduces assay sensitivity due to genetic heterogeneity.

***Optional:*** We tested a microfluidic-based single cell sorter, yet the limiting dilution was as efficient as the single cell sorter and has an easier and less expensive workflow.

***Note:*** We observed the plate at the same time every day from day 7^th^ to 14^th^ after single cell isolation to ensure that all selected clones are monoclonal and growing at the same rate.

4. Expand the monoclonal colonies on the 14^th^ day post isolation.
  a. Wash the wells that contain monoclonal colonies twice with 1X PBS.
  b. Trypsinize the monoclonal colonies in 100 μL of 0.25% trypsin-EDTA and incubate at 37°C for 5 min.
  c. Add 100 μL of media and break up all cell clumps into individual cells by pipetting up and down.
  d. Transfer 200 μL of the cell-containing media to a single well in a 12-well plate.
  e. Add 800 μL of media to each well containing cells in a 12-well plate.

***Note:*** We noticed that some colonies are not easily detached during trypsinization from a 96-well plate. To minimize cell loss, make sure to check cells under a microscope to determine whether they are fully detached.

5. Once the monoclonal colonies in the 12-well plate are confluent, collect the cells.
  a. Wash the cells once with 1X PBS.
  b. Trypsinize in 500 μL of 0.25% trypsin-EDTA (enough to cover the cells) and incubate at 37°C for 5 min.
  c. Add 500 μL of media (≥ volume of trypsin added) and break up all cell clumps into individual cells by pipetting up and down.

### Cryopreservation of monoclonal cell lines

6. Select a few monoclonal cell lines from above and expand the cells by placing each individual monoclonal cell line in a separate T-25 flask.
7. Add 5 mL of media and incubate at 37°C until the cells are confluent.
8. Freeze the cells at 1.5 × 10^6^ cells per cryovial in 1 mL of media containing 5% DMSO.
9. Store the cells at −80°C.

**CRITICAL:** The purpose of this step is to preserve monoclonal colonies that are genetically homogeneous. Pre-existing genetic heterogeneity will lead to an overestimation in array change. The frozen stocks will be used to initiate clonal evolution.

### Preparation of HOR CN control

10. Centrifuge the remaining parent cells after single-cell isolation at 300 × *g* for 5 min.
11. Remove the supernatant.
12. Store the parent cell pellet at −20°*C until the* droplet digital PCR (*ddPCR) run is needed.*

### Preparation of positive control

13. Thaw a CHM13 cryostock containing 2 × 10^6^ cells and expand the cells to 10^7^ cells.
14. Add 10^6^ cells in each 1.5 mL microcentrifuge tube.
15. Centrifuge the cells at 200 × *g* for 5 min.
16. Remove the supernatant.
17. Store the cell pellet at −20°*C until the ddPCR run is needed.*

### Preparation of 20X primer-pair working stock

18. Prepare a 20X primer-pair working stock for the targeted HOR and reference single gene (refer to Materials and equipment setup below).
19. Store the 20X working stock at −20°*C*.

## Key resources table

**Table:** 

REAGENT or RESOURCE	SOURCE	IDENTIFIER
**Chemicals, peptides, and recombinant proteins**		

AluI	New England Biolabs	CAT#R0137
HaeIII	New England Biolabs	CAT#R0108
Fetal bovine serum (FBS)	Cytiva	CAT#SH3007003
Iscove’s modified Dulbecco’s medium (IMDM)	ATCC	CAT#30-2005
Antibiotic-Antimycotic	Gibco	CAT#15240062
DMEM, high glucose, GlutaMAX	Gibco	CAT#10569010
AmnioMAX C-100 supplement	Gibco	CAT#12556023
AmnioMAX C-100 basal medium	Gibco	CAT#17001074
Penicillin-Streptomycin	Gibco	CAT#15140122
Buffer elution buffer	QIAGEN	CAT#79217
Dulbecco’s phosphate-buffered saline (PBS) 1X	Sigma-Aldrich	CAT# D8537
0.25% Trypsin/EDTA	Thermo Fisher Scientific	CAT#25200056
Dimethyl sulfoxide (DMSO)	Sigma-Aldrich	CAT#41640-100ML

**Critical commercial assays**		

DNeasy Blood & Tissue Kit	QIAGEN	CAT#69504
Ultra-high sensitivity assay	DeNovix	DSDNA-ULTRA-2
High sensitivity dsDNA assay	DeNovix	DSDNA-HIGH-1
Qx200 ddPCR EvaGreen Supermix	Bio-Rad	CAT#1864034
Automated droplet generation oil for EvaGreen	Bio-Rad	CAT#1864112
ddPCR droplet reader oil	Bio-Rad	CAT#1863004

**Experimental models: cell lines**		

Human: U2OS	ATCC	HTB-96
Human: K562	ATCC	CCL-243
Human: CHM13	Magee-Womens Hospital (Pittsburgh, PA)	N/A

**Oligonucleotides**		

Chromosome-specific HOR/single gene primers	Integrated DNA Technologies	Table 1

**Software and algorithms**		

RStudio	RStudio: Integrated Development for R. RStudio, PBC, Boston, MA	https://posit.co/downloads/
QuantaSoft version 1.7	Bio-Rad	CAT#186-4011

**Other**		

96-well plates	Fisher Scientific	CAT#3894
12-well plates	Fisher Scientific	CAT#07-200-83
T-75 flasks	Fisher Scientific	CAT#13-680-65
T-25 flasks	Fisher Scientific	CAT#07-000-225
EMD Millipore Steriflip sterile disposable vacuum filter units (0.22 μm)	Fisher Scientific	CAT#SCGP00525
Pipette tips RT LTS 200 μL	Rainin	CAT#30389240
Pipette tips RT LTS 1000 μL	Rainin	CAT#30389212
Pipette tips RT LTS 20 μL	Rainin	CAT#30389226
Falcon 50 mL conical centrifuge tubes	Fisher Scientific	CAT#14-432-22
Falcon 15 mL conical centrifuge tubes	Fisher Scientific	CAT#14-959-49B
Corning internally threaded cryogenic vials	Fisher Scientific	CAT# 03-374-20
ddPCR 96-well plates, semi-skirted	Bio-Rad	CAT#12001925
PCR plate heat seal, foil, pierceable	Bio-Rad	CAT#1814040
PX1 PCR plate sealer	Bio-Rad	CAT#181-4000
C1000 Touch Thermal cycler with 96-deep well reaction module	Bio-Rad	CAT#1840197
QX200 droplet reader	Bio-Rad	CAT#1864001
Automated droplet generator	Bio-Rad	CAT#1864101
DG32 automated droplet generator cartridges	Bio-Rad	CAT#1864108
Pipet tips for the AutoDG system	Bio-Rad	CAT#1864120
Pipet tip waste bins for the AutoDG system	Bio-Rad	CAT#1864125
Vi-CELL BLU cell viability analyzer	Beckman Coulter Life Sciences	CAT# C19201
Qubit 3.0 Fluorometer (with DeNovix Ultra High Sensitivity Assay.qbt file)	Thermo Fisher Scientific	CAT#Q33216
Leica DMi1 inverted microscopes	VWR	CAT#10752-270

## Materials and equipment

This protocol uses the QX200 Droplet Digital PCR system which includes an automated droplet generator, PX1 PCR plate sealer, thermal cycler with 96–deep well reaction module, and QX200 droplet reader which comes with QuantaSoft Software (version 1.7). QuantaSoft is compatible with Windows 10 operating systems. The Droplet Digital PCR Applications Guide is available from the Bio-Rad website: https://www.bio-rad.com/webroot/web/pdf/lsr/literature/Bulletin_6407.pdf.

**Table:** 20X primer-pair working stock

Reagent	Final concentration	Amount
100 μM forward primer	2 μM	4 μL
100 μM reverse primer	2 μM	4 μL
ddH_2_O	N/A	192 μL
**Total**	**N/A**	**200 μL**

Store 20X primer-pair working stock at −20°C.

**Table:** Droplet Digital PCR (ddPCR) reaction mix

Reagent	Final concentration	Amount
20X Primer pair stock (100 μM)	1X	1 μL
Alu I	2 units/mL	0.2 μL
2x ddPCR supermix for EvaGreen	1X	10 μL
DNA template	N/A	1 μL
ddH_2_O	N/A	7.8 μL
**Total**	**N/A**	**20 μL**

Prepare the reaction mix immediately before conducting the experiment and store on ice.

**CRITICAL:** The fresh master reaction mix without the DNA template should be made just before the run. The gDNA of the samples will be added individually.

**CRITICAL:** Vortex all reagents and spin down before preparing the reaction. When preparing the ddPCR reaction mixture, add 1.25 x the total required volume because the viscosity of 2X supermix may lead to a pipetting error (see Figure 2 Column 6). Prepare a reaction in multiples of 8 because the automatic droplet generator takes 8 samples (1 column of a 96-well plate) and generates droplets simultaneously.

## Step-by-step method details

### Isolation of subclones from a monoclonal cell line for clonal evolution

1. Isolate subclones as described in the steps 1–5 of the monoclonal cell line preparation section above using the monoclonal cell line frozen stock. Troubleshooting 1.
2. Place the cells in 1.5 mL microcentrifuge tubes.
3. Centrifuge at 300 × *g* for 5 min.
4. Remove the supernatant.
5. Store the cell pellets of subclones at −20°*C until the ddPCR run is needed.*

### Genomic DNA extraction from subclones and controls

**Timing: 30 min. Time depends on the number of samples**

6. Thaw frozen sub-clone cell pellets, parent cells, and ddPCR positive control that are stored at −20°*C*.
7. Resuspend pellets in 200 μL of 1X PBS.
8. Using the QIAGEN blood and tissue kit, add 20 μL of proteinase K.
9. Add 200 μL Buffer AL and mix thoroughly by vortexing.
10. Add 200 μL of ice-cold 100% ethanol and mix thoroughly.
11. Place the mixture into a DNeasy mini spin column and centrifuge at >6,000 × *g* for 1 min.
12. Discard the flowthrough.
13. Add 500 μL Buffer AW1 and centrifuge at >6,000 × *g* for 1 min.
14. Discard the flowthrough.
15. Add 500 μL Buffer AW2 and centrifuge at >20,000 × *g* for 1 min.
16. Discard the flowthrough.
17. Centrifuge at >20,000 × *g* for 3 min.
18. Discard the flowthrough.
19. Transfer the column to a new 1.5 mL microcentrifuge tube.
20. Add 200 μL of Buffer AE to the center of the column and incubate at room temperature for 1 min.
21. Insert columns into tubes and centrifuge at >6,000 × *g* for 1 min.
22. Discard the column and collect the gDNA.

***Note:*** We tested a direct PCR lysis reagent and NucleoSpin tissue XS assay to reduce the number of cells needed, but the results using these assays were not as consistent as the results from the QIAGEN blood and tissue kit.

**Pause point:** gDNA can be stored at −20°*C*.

### Genomic DNA quantification and dilution

**Timing: at least 30 min. Required time depends on the number of samples**

23. Measure the concentrations of the gDNA extracts from the cell pellets using a DeNovix dsDNA high sensitivity assay.
  a. Equilibrate all components to room temperature before use.
  b. Vortex and spin down all components.
  c. Prepare a working solution for the samples and two standards. Each standard and unknown sample requires 190 μL of working solution. Add 1.9 μL of 100X dye in the 188.1 μL buffer per measurement. Scale up as needed to make enough volume to use.
  d. Vortex to mix thoroughly.
  e. Aliquot 190 μL of the working solution in a thin, UV-transparent 0.5 mL tube for each sample and standard.
  f. Add 10 μL of standard and unknown sample to the assay tubes.
  g. Vortex tubes to mix.
  h. Incubate the samples and standards at room temperature for 5 min.
  i. Generate a standard curve by DeNovix DS-11 FX using two standards.
  j. Measure the concentration of the samples.

***Optional:*** any assay that can quantify 100 pg–250 ng range fluorometrically including Qubit dsDNA assays can be used.

24. Vortex thoroughly and dilute samples to 2 ng/μL in Buffer AE using Rainin low retention tips.
25. Measure the concentration of 2 ng/μL stock using a DeNovix dsDNA ultra-high sensitivity assay.
  a. Equilibrate all components to room temperature before use.
  b. Vortex and spin down all components.
  c. Prepare a working solution for the samples and two standards. Each standard and unknown sample requires 200 μL of working solution. Add 0.5 μL of the 400X dye and 2 μL of the 100X enhancer in 197.5 μL buffer per measurement. Scale up as needed to make enough volume to use.
  d. Vortex to mix thoroughly.
  e. Aliquot 200 μL of the working solution in a thin, UV-transparent 0.5 mL tube for each sample and standard.
  f. Add 10 μL of standard and unknown sample to the assay tubes.
  g. Vortex tubes to mix.
  h. Incubate samples and standards at room temperature for 5 min.
  i. Generate a standard curve by DeNovix DS-11 FX using two standards.
  j. Measure the concentration of the samples.
26. Repeat steps 24–25 until 2 ng/μL (< ± 10%) stocks are made from all samples.
27. Vortex thoroughly and dilute 2 ng/μL stock to 1:20 in Buffer AE using Rainin low retention tips.

**CRITICAL:** The CN of the reference single gene is used to normalize the CN of the HOR of the same subclone. To minimize subsampling error, it is important to make the 2 ng/μL stock as accurately as possible and mix thoroughly before preparing the diluted gDNA stock.

**Pause point:** Diluted gDNA can be stored at −20°C.

### ddPCR reaction setup

**Timing: 1 h (96 samples)**

**CRITICAL:** We measure the HOR and reference single gene (SG) CNs in separate reactions using the EvaGreen assay due to constraints caused by the differences in ddPCR dynamic range between the HOR and reference SG when using the same amount of gDNA input. Therefore, we use 2 ng of gDNA for the reference SG measurement and a 1:20 dilution of 2 ng gDNA stock for HOR measurement. This is the optimal condition to quantify D11Z1 CN in U2OS cells. The optimal gDNA concentration for each different SG and dilution factor for each different HOR should be determined based on the CN of target HOR and SG in the cell of interest to fit within the dynamic range of the ddPCR.

**CRITICAL:** EvaGreen dye binds to both double-stranded DNA and nonspecifically single-stranded DNA. Therefore, concentrations of gDNA input and primer need to be optimized to ensure accurate CN measurement.

**CRITICAL:** To minimize subsampling error, use low retention tubes and tips for all steps and pre-wet tips to avoid bubbles in the tips.

**CRITICAL:** Avoid introducing air bubbles to the droplet generator cartridge. This will impact droplet generation and reduce the assay efficiency. Therefore, add 10% excess volume (22 μL) to prevent air bubble formation (see Figure 2. Column 4).

28. Thaw the diluted gDNA stocks of subclones, parental cells, and ddPCR control.
29. Vortex thoroughly to homogenize gDNA in solution.
30. Place 2X supermix and 20X primer pair stocks at room temperature to thaw.
31. Following the ddPCR reaction mix table in the materials and equipment section above, prepare two ddPCR master reaction mixes without gDNA (the gDNA will be added in step 35) according to the number of samples needed, in triplicate (see Figure 2. Column 5) and including the two controls.

**Figure 1:**
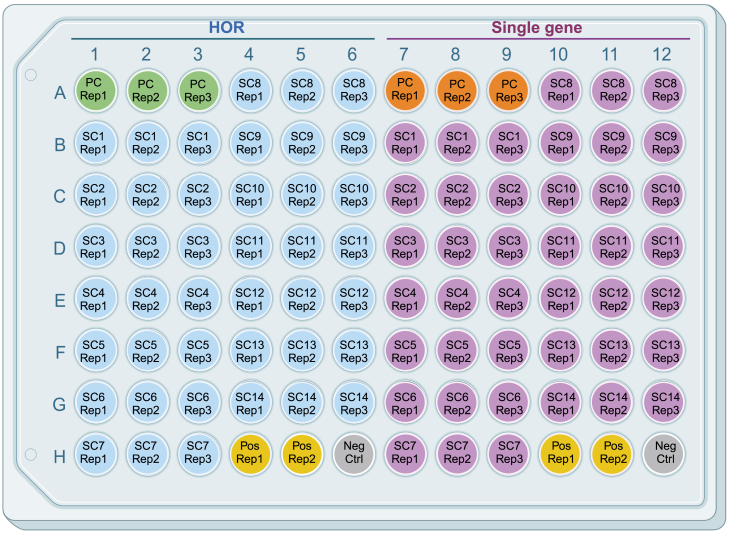
Example plate layout for quantifying copy numbers of higher order repeats (HOR) and reference single gene (SG) across subclones in a 96-well plate In columns 1–6 the HOR measurements are highlighted blue and in columns 7–12 the reference SG are highlighted purple. The parental cells (PC) that provide the baseline HOR and SG CN are highlighted green and orange respectively. The assay positive control is highlighted yellow. All measurements are in triplicate except the assay positive control (yellow) and the no-template control (gray).

**Figure 2:**
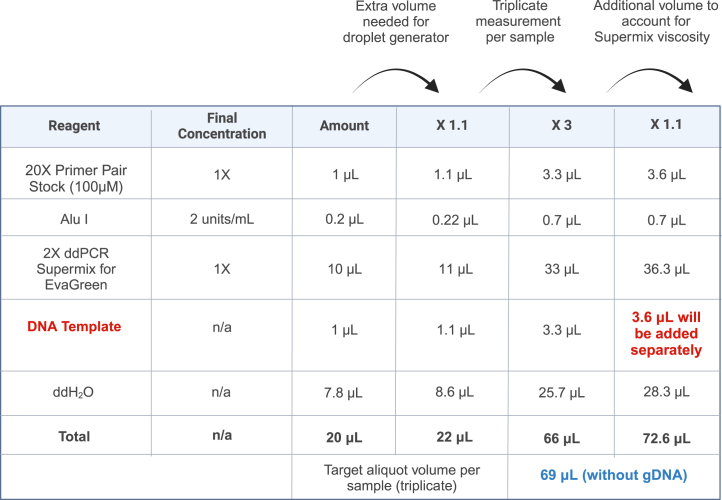
An example volume calculation for the reaction master mix Additional master mix should be prepared to prevent bubble formation (1.1x the amount used for a single reaction), to account for triplicate measurement (3x), and Supermix viscosity (1.1x the triplicate volume). The gDNA template (red) will be added separately. The target volume for each sample in triplicate is at least 69 μL (blue). See also Figure 1 and Table 1.

***Note:*** One master mix will include the HOR target primers and the other will include the single gene reference primers.

***Note:*** Refer to Figure 1 for a suggested plate layout and Figure 2 for an example of the master mix volume calculation.

**CRITICAL:** Incubate the ddPCR reaction with the restriction enzyme for at least 30 min to ensure every CN of the HOR and SG are isolated and partitioned into droplets.

32. Label 1.5 mL low retention microcentrifuge tube that match the number of samples that are being measured.
33. Vortex thoroughly and aliquot 69 μL of reaction master mix to each tube.
34. Vortex thoroughly to mix the gDNA.
35. Add 3.6 μL of either 2 ng/μL (SG), 1:20 dilution of the 2 ng/μL stock (HOR), or ddH_2_O to the corresponding tubes containing the target primer-pair.
36. Vortex thoroughly to mix the reaction.
37. Incubate the reaction in the dark for 30 min.
38. Vortex tubes thoroughly to homogenize the reactions.
39. Aliquot 22 μL from each tube to each corresponding well in a 96-well ddPCR plate in triplicate (see Figure 1).
40. Turn on the PCR plate sealer which is set to seal at 180°C for 5 s.
41. Place a pierceable foil seal on the top of the plate (the redline should be on the top of the plate) and seal the plate.
42. Vortex the plate at 2000 rpm for 1 min.
43. Centrifuge the plate at 1000 × *g* for 1 min.

**CRITICAL:** Remove air bubbles and ensure the ddPCR reaction mix is at the bottom of the wells. If there are any bubbles after centrifugation, repeat step 42.

### Droplet generation

**Timing: ∼45 min (for 96 samples). It will take less time for fewer samples. It takes an average of 3.5 min per column**

44. Set up the configuration on the automated droplet generator (AutoDG) as follows.
  a. Select oil type as EvaGreen.
  b. Specify the columns that contain the samples.
45. Replace the oil and consumables as follows (the green lights on the instrument decks or touchscreen will turn on if the consumables are placed correctly).
  a. Ensure that the oil volume is sufficient and the type of droplet generation oil is EvaGreen.
  b. Load droplet generator cartridges onto the cartridge blocks on the back row (the green gaskets should be on the right side).
  c. Insert an empty pipet tip waste bin.
  d. Remove the lids and place pipet tips on the pipet tip blocks on the middle row.
  e. Place a 96-well ice cold chill block on the droplet plate holder (front right).
  f. Place a new ddPCR 96-well plate in the 96-well chill block.
46. Insert the sealed ddPCR reaction plate into the sample plate holder (front left).
47. Start the AutoDG when all requirements are satisfied.
48. Once the droplet generation is complete, place a pierceable foil seal onto the 96-well plate containing the droplets in the chill block (the red line should be on the top of the plate).
49. Gently remove the plate and seal at 180°C for 5 s.

**CRITICAL:** The droplets are very fragile. We recommend processing the PCR amplification step as soon as the droplet generation is complete. Do not vortex or spin down the plate. Handle the plate very carefully until it is loaded on the thermal cycler.

**CRITICAL:** Improper sealing of the plate leads to oil evaporation during thermal cycling and can compromise droplet data quality.

***Note:*** See the AutoDG instrument manual for details: https://www.bio-rad.com/webroot/web/pdf/lsr/literature/10043138.pdf

***Note:*** A QX200 droplet generator can be used instead of the AutoDG. See the instrument manual for details: https://www.bio-rad.com/webroot/web/pdf/lsr/literature/10031907.pdf

***Note:*** Droplets will have an opaque layer at the top of each well if the droplets are properly generated.

***Note:*** The chill block needs to be stored at −20°C upside down for more than 2 h before use. The instrument is not sensitive enough to detect errors occurring in this block such as a misplaced sample plate or a failure to place a new plate. A mistake in this step can lead to the failure of the entire run.

***Note:*** The completion time for droplet generation can vary between runs. We recommend monitoring the instrument performance until the first column is processed before leaving the machine.

### Thermal cycling (PCR)

**Timing: 2.5 h**

50. Transfer the sealed droplet plate to a thermal cycler and start the PCR cycling using the program as below (Table 2). The volume of the thermal cycling reaction is 40 μL. The ramp rate should be 2°C/s for all cycles.

**Table 2:** PCR cycling conditions

Steps	Temperature	Time	Cycles
Enzyme activation	95°C	10 min	1
Denaturation	96°C	30 s	40 cycles
Annealing/extension	56°C	60 s	
Signal stabilization	4°C	5 min	1
	90°C	5 min	1
Hold	4°C	Forever

**CRITICAL:** The annealing temperature must be optimized for every set of primers using a temperature gradient (65°C–55°C).

***Note:*** We noticed that incubating the plate at 4°C overnight after PCR cycling increases the number of accepted droplets.

**Pause point:** The plate can be stored at 4°C overnight after completion of the PCR step until droplet reading.

### Droplet reading

**Timing: 2 h (for 96 samples). Droplet reading takes about 10 min for each column**

51. Remove the metal retainer and insert the sealed PCR-amplified reaction plate into the QX200 droplet reader and secure the retainer.
52. Open the Quantasoft software and prime the QX200 droplet reader.
53. Set up a new plate layout according to the experimental samples.
54. Designate the parameters as follows.
  a. Define “Supermix” as “EvaGreen”
  b. Set “Experiment Type” as “Direct Quantification”
  c. Assign “Assay Type” as “Single Target per Channel”.
  d. Under “Target Info”, select signal Ch1 as “EvaGreen” and signal Ch2 as “None”
  e. Configure the names of the samples.
55. Start the ddPCR reading.
56. Once the reading is complete, remove the empty plate from the QX200 droplet reader and dispose the waste in accordance with institutional, state, and local regulations.

***Note:*** ddPCR droplet reading will not proceed if the droplet reading oil is insufficient for the reaction or the waste container is full.

## Expected outcomes

Successful completion of the monoclonal isolation step should result in greater than 25 monoclonal cells, with an average of 4 doublets, when approximately 0.5 cells/well was added to the 96-well plate. A successful ddPCR run should result in >16,000 accepted droplets and a separation of two peaks representing the amplitudes of the negative (background) and the positive (target). The CN of D11Z1 in the positive control (CHM13) should be ∼3,500 copies/μL with minimal background in the negative control.

## Quantification and statistical analysis

### Overview

The purpose of this section is to quantify the absolute number of copies/μL for both HOR and SG and normalize the HOR CN by SG CN to estimate an average of HOR CN per centromeric array. The HOR CN per array across the subclones will be compared to the value of parental monoclonal cells that the subclones were isolated from. The frequency of CN change across the subclones is determined using an ANOVA test followed by Tukey’s Honestly Significant Difference test. The two steps that are needed are as follows.

 Use Quantasoft to estimate the CN.

 Quantify HOR CN per array by normalizing with the SG CN.

The number of accepted droplets and negative droplets are used to calculate # copies/μL. The number of positive droplets influences the technical error of the measurement.

### Data analysis

**Timing: 0.5 h**

**Figure 3:**
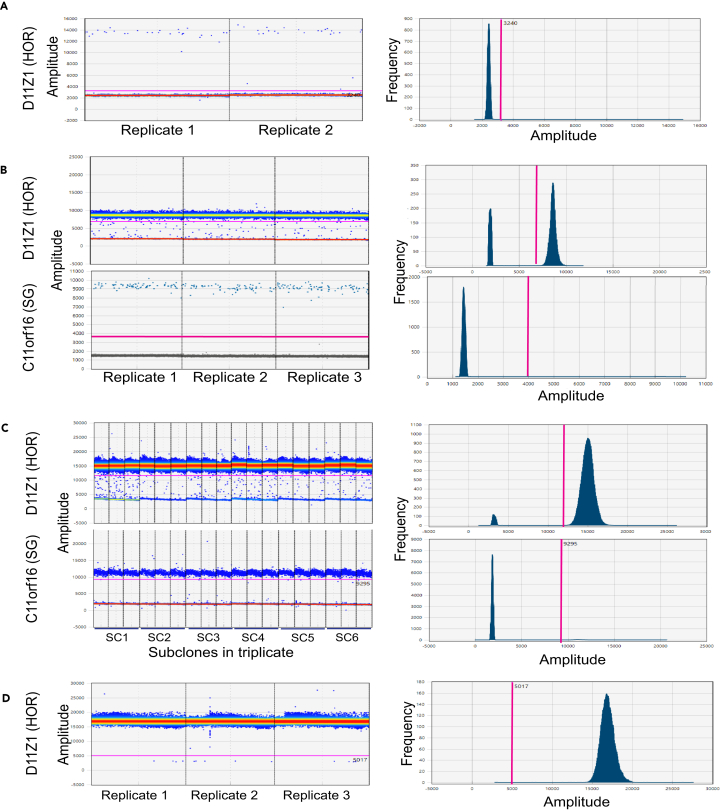
Examples of ddPCR analysis using Quantasoft (A) Example of horizontal and vertical gating to separate the negative droplets from the no-template control targeting D11Z higher order repeats (HOR) in two technical replicates. The pink line represents the amplitude threshold that is used to distinguish between negative and positive droplets. The blue dots above the pink line are positive droplets. (B) Gating example for HOR and reference single gene (SG) in the positive control CHM13. (C) Gating example for HOR and reference SG across six subclones of U2OS in triplicate. (D) Example of quantification failure due to excess gDNA input.
3. Determine the amplitude threshold to separate the positive and negative droplets for the purpose of quantification as follows.
  a. Select all experimental wells targeting HORs.
  b. Click the 1-D amplitude plot view.
  c. Manually place a horizontal gate to separate the clusters of positive and negative droplets.
  d. Repeat steps a-c with all wells targeting SG.
  e. Export the results.csv file from Quantasoft.

***Note:*** For a more conservative approach for defining positive droplets, set the horizontal gate close to the main cluster of positive droplets.

1. After completion of the droplet reading, open Quantasoft and select the run.
2. Select the well that contains the NTC and ensure the well is free of contamination (see Figure 3 and Troubleshooting 2).

4. To further increase data quality, open the results.csv file and exclude any well that meets the following criteria.
  a. < 10,000 accepted droplets.
  b. A distinctively different average amplitude from the wells that share the same target.
  c. Number of positive droplets are < 100.
  d. Number of negative droplets are < 10.
5. Multiply the HOR CN x 20 to account for the dilution factor difference between the HOR and SG gDNA input (1:20).
6. Normalize the HOR CN with the SG CN by pairing the first HOR and SG replicates and dividing the HOR CN by the SG CN.
7. Repeat step 6 for any other technical replicates.
8. Conduct the ANOVA test followed by Tukey’s HSD test between each subclone CN and the parental CN value in R.

## Limitations

Due to single-cell isolation requirement, this protocol requires four weeks to collect the subclones for measurement. Therefore, the protocol is limited to cell lines that can grow after single cell isolation. A lower number of cell divisions may increase the sensitivity of the CN change detection due to sharing higher genetic homogeneity in monoclonal populations. However, 5 × 10^5^ cells were required for gDNA extraction to yield consistent results with less technical error. Since we used previously published and tested primers,^1^ no primer design step is included in this protocol.

## Troubleshooting

### Problem 1

A high number of doublets are observed after monoclonal isolation in step 1.

### Potential solution

Use a 40 μm cell strainer mesh instead of breaking up clumps by pipetting. Add fewer than 50 cells/96-well and isolate the monoclonal cells in two 96-well plates to collect enough subclones.

### Problem 2

Positive droplets observed in the NTC in step 2 (see Figure 3A).

### Potential solution

Due to the exceptionally high CN of HORs and sensitivity of the ddPCR, any cross-contamination between samples during ddPCR preparation can cause signal amplification. We recommend cleaning the pipettes and workbench before beginning the assay and use filter tips to prevent any contamination.

### Problem 3

Read failure or presence of extremely high copies reported in Quantasoft (see Figure 3D).

### Potential solution

Inaccurate gDNA quantification can lead to an excess amount of gDNA input. This may result in the ddPCR creating very few or even no negative droplets, resulting in read failure or an extremely high reported CN. We recommend re-quantifying the input or titrating the gDNA and running a test plate to determine the optimal gDNA input to fit within the ddPCR dynamic range.

### Problem 4

The CN in the positive control is lower than the expected value.

### Potential solution

CN measurement can be low when the restriction enzyme digestion is incomplete, resulting in partial isolation of target copies. This can be caused by an insufficient amount of enzyme, an unsatisfactory enzyme, or insufficient incubation time. We recommend using a fresh vial of enzyme and incubate at least 30 min.

### Problem 5

Inconsistent CN between replicates.

### Potential solution

High subsampling error can contribute to inconsistent CN between replicates. One of the major sources of high subsampling error is a low number of positive droplets. The coefficient of variation is 6% (14.6 copies/μL) and 10% (5.8 copies/μL) when the positive droplets are below 250 and 100 respectively with an assumption of 20,000 accepted droplets. We recommend discarding measurements that are below 100 positive droplets.

## Resource availability

### Lead contact

Further information and requests for resources and reagents should be directed to and will be fulfilled by the lead contact, Dr. Steven Henikoff (steveh@fredhutch.org).

### Technical contact

Questions about the technical specifics of performing the protocol should be directed to the technical contact, Dr. Steven Henikoff (steveh@fredhutch.org).

### Materials availability

This study did not generate any new unique reagents.

### Data and code availability

This study did not generate new datasets in this study. The published article^1^ includes all datasets generated in the study.
